# Application of analgesics in emergency services in Germany: a survey of the medical directors

**DOI:** 10.1186/s12873-023-00878-8

**Published:** 2023-09-14

**Authors:** Signe Vilcane, Olga Scharonow, Christian Weilbach, Maximilian Scharonow

**Affiliations:** 1 Department of Anaesthesiology, Intensive Care Medicine, Emergency Medicine and Pain Therapy, St. Josefs-Hospital Cloppenburg, Academic Teaching Hospital of the Hannover Medical School (MHH), Krankenhausstrasse, 13, 49661 Cloppenburg, Germany; 2 Department of Internal Medicine, St. Josefs-Hospital Cloppenburg, Academic Teaching Hospital of the Hannover Medical School (MHH), Cloppenburg, Germany

**Keywords:** Analgesia, Administration and dosage, Prehospital emergency care, Medical directors

## Abstract

**Background:**

Treatment of acute pain is an essential element of pre-hospital care for injured and critically ill patients. Clinical studies indicate the need for improvement in the prehospital analgesia.

**Objective:**

The aim of this study is to assess the current situation in out of hospital pain management in Germany regarding the substances, indications, dosage and the delegation of the use of analgesics to emergency medical service (EMS) staff.

**Material and methods:**

A standardized survey of the medical directors of the emergency services (MDES) in Germany was carried out using an online questionnaire. The anonymous results were evaluated using the statistical software SPSS (Chi-squared test, Mann-Whitney-U test).

**Results:**

Seventy-seven MDES responsible for 989 rescue stations and 397 EMS- physician bases in 15 federal states took part in this survey. Morphine (98.7%), Fentanyl (85.7%), Piritramide (61%), Sufentanil (18.2%) and Nalbuphine (14,3%) are provided as opioid analgesics. The non-opioid analgesics (NOA) including Ketamine/Esketamine (98,7%), Metamizole (88.3%), Paracetamol (66,2%), Ibuprofen (24,7%) and COX-2-inhibitors (7,8%) are most commonly available. The antispasmodic Butylscopolamine is available (81,8%) to most rescue stations.

Fentanyl is the most commonly provided opioid analgesic for treatment of a traumatic pain (70.1%) and back pain (46.8%), Morphine for visceral colic-like (33.8%) and non-colic pain (53.2%). In cases of acute coronary syndrome is Morphine (85.7%) the leading analgesic substance. Among the non-opioid analgesics is Ketamine/Esketamine (90.9%) most frequently provided to treat traumatic pain, Metamizole for visceral colic-like (70.1%) and non-colic (68.6%) as well as back pain (41.6%). Butylscopolamine is the second most frequently provided medication after Metamizole for “visceral colic-like pain” (55.8%).

EMS staff (with or without a request for presence of the EMS physician on site) are permitted to use the following: Morphine (16.9%), Piritramide (13.0%) and Nalbuphine (10.4%), and of NOAs for (Es)Ketamine (74.1%), Paracetamol (53.3%) and Metamizole (35.1%). The dosages of the most important and commonly provided analgesic substances permitted to independent treatment by the paramedics are often below the recommended range for adults (RDE). The majority of medical directors (78.4%) of the emergency services consider the independent application of analgesics by paramedics sensible. The reason for the relatively rare authorization of opioids for use by paramedics is mainly due to legal (in)certainty (53.2%).

**Conclusion:**

Effective analgesics are available for EMS staff in Germany, the approach to improvement lies in the area of application. For this purpose, the adaptations of the legal framework as well as the creation of a guideline for prehospital analgesia are useful.

## Background

The management of pain is an important part of prehospital care of ill or injured patients. From clinical studies, There is a need to improve analgesia in the ambulance service [1–3].

A medical director of the emergency services (MDES) is responsible for the implementation of medical standards, following regional protocols. This individual is appointed by the cities / districts in accordance with rescue laws of the respective federal state. One of their responsibilities is the selection of substances and methods for analgesic treatment in emergency use. Another part of this is the delegation of medical procedures to emergency medical service (EMS) staff i.e. paramedics, who since the ‘Paramedic Law’ of 2014 are allowed to carry out “medical measures” according to regionally defined rules [4].

From a legal point of view, treatment with non-opioid analgesics is easier to delegate than treatment with opioids, which is carried out according to the Narcotics Act. The administration of opioids, however, was a subject to the provisions of the German Federal Narcotics Act. The amendment of the German Federal Narcotics Act allows the treatment with opioids also by emergency paramedics within a defined framework from August 2023 [4, 5].

In recent years, several papers have been published on the application of strong analgesics by paramedics [6–16]. With increasing possibilities in the field of telemedicine, this topic was also accompanied in the context of scientific projects and studies [17, 18].

There is little knowledge about the implementation of the evidence obtained in the regional algorithms regarding delegation and application of strong analgesics to the paramedics in Germany; the same applies to out of hospital analgesia in general.

By carrying out a survey, and using the MDES as participants, we will be able to investigate the substances and combinations of analgesics that are used to treat pain by physicians and paramedics in Germany. In addition, the different indications, dosage and training procedures, as well as the subjective evaluations, are the focus of this study.

## Materials and methods

The current open questions in the field of prehospital analgesia were recorded within the framework of a literature search and on the basis of our own experience.

During the test phase, the initial questionnaire was distributed to the MDES of eight neighboring EMS areas, and reviews and suggestions were incorporated into the final form of the questionnaire. The final questionnaire consisted of a total of 28 questions.

The questionnaire was created and processed using the open source software "LimeSurvey", which was installed on a domain registered for this purpose. The questionnaire could be accessed via a link generated and activated for this purpose. The possibility of multiple participations by the same person was circumvented by setting cookies.

Medical directors of the emergency services in Germany were invited via email to take part in the survey. A reminder email was sent at an interval of 5 weeks. After a total period of 10 weeks, we deactivated access to the domain, which concluded the active survey phase.

Anonymization and statistical analysis of the collected data were performed. In addition to the location-related data, the influence of the size and structure (rural/urban…) of the ambulance service area, the ambulance service provider, the reasons for or against delegation of analgesia on the algorithms/therapy options was investigated using the Chi^2^ test, the influence of the number of ambulance stations or EMS- physician bases on the approval of independent therapy was investigated using the Mann–Whitney-U-Test by means of the statistical software SPSS version 24.

The survey was carried out following approval of the Ethics Committee of the University Medical School Hannover (No. 9276_BO_K_2020).

## Results

### Location-related data

Seventy-seven MDES responsible for 989 EMS stations *(M 13,2 / SD 10,9)* and 397 EMS- physician locations *(M 5,2/ SD 3,5)* in 15 federal states took part in this survey.

More than half of the participants (59.8%) work in a rural ambulance service area (14.3% with a population density < 100 inhabitants/km^2^ and 45.5% with 100 to 150 inhabitants/km^2^), 27.1% in urban and 13% in mixed ambulance service areas (several counties including large cities).

The authorities as bodies responsible for the emergency services were distributed as follows: districts (64.9%), cities (20.8%) and mixed providers (14.3%).

The considerable differences between the scope of leadership of the MDES were mainly dependent on the ambulance service structures of the different federal states.

In most EMS areas more than 20,000 EMS callouts (77.9%) and over 1,500 EMS physician callouts (80.5%) are performed each year.

In terms of EMS vehicles, both emergency ambulances (EA) and emergency physician vehicles (EPV) are stationed in all 77 rescue service areas. Rescue helicopters (31.2%), intensive care transport vehicles (28.6%), non-critical patient carriers (N-KTWs) (24 0.7%) and ambulance with emergency physician (11.7%) are also available. The "rarer rescue vehicles" include obesity ambulance vehicles (6,5%), tele-emergency physician (2,6%), intensive care helicopter, pediatric and newborn emergency physician service (each 1,3%).

### Analgesics, forms of application, indications

In terms of drug equipment, in the opioid analgesic group, morphine (98.7%) is available in almost every ambulance service area, followed by fentanyl (85.7%), piritramide (61%), sufentanil (18.2%), and nalbuphine (14.3%). The opioid analgesics tramal and buprenorphine (1.3% each) are rarely used.

Of the non-opioid analgesics, (es)ketamine (98.7%), metamizole (88.3%), paracetamol (66.2%), ibuprofen (24.7%), and COX-2 inhibitors (7.8%) are most commonly present. The spasmolytic agent butylscopolamine is also a commonly available agent for analgesic therapy at 81.8%.

The routes of administration approved by the MDES and the indications for the use of opioid and non-opioid analgesics are summarized in Table 1.

**Table 1:** Substances, forms of application and indications

Analgesics	Methods of administration						Indications				
	i.v.	i.m.	s.c.	nas.	oral	rec.	T	V	C	A	B
Fentanyl (*n* = 66/85,7%) *R* = 844/85,3%	•	•	•	•	•		•	•	•	•	•
Morphine (*n *= 76/98,7%) *R *= 987/89,7%	•	•	•	•			•	•	•	•	•
Piritramide (*n *= 47/61%) *R *= 574/58,0%	•	•	•	•			•	•	•	•	•
Sufentanil (*n *= 14/18,2%) *R *= 290/29,3%	•	•	•	•			•	•	•	•	•
Nalbuphine (*n* = 11/14,3%) *R* = 130/13,1%	•	•	•	•			•	•	•	•	•
Buprenorphine (*n* = 1/1,3%) *R* = 11/1,1%	•	•	•				•	•	•		•
Tramadol (*n* = 1/1,3%) *R* = 11/1,1%	•										•
Ketamine (*n* = 76/98,7%) *R* = 987/99,8%	•	•	•	•	•	•	•	•			•
Paracetamol (*n* = 51/66,2%) *R* = 669/67,6%	•	•			•	•	•	•			•
Metamizole (*n* = 68/88,3%) *R* = 865/87,5%	•				•		•	•			•
COX-2-inhibitors (*n* = 6/7,8%) *R* = 90/9,1%	•				•		•				•
Ibuprofen (*n* = 19/24,7%) *R* = 305/30,8%					•	•	•	•			•
Diclofenac (*n* = 1/1,3%) *R* = 6/0,6%							•				•
Butylscopolamine (*n* = 63/81,8%) *R* = 846/85,5%	•							•	•		

*i.v.* intravenous, *i.m.* Intramuscular, *s.c.* Subcutaneus, *nas.* Nasal, *rek.* Rectal

*R* Number of rescue stations

*T* Traumatic pain, *V* Visceral non-colic pain, *C* Colic pain, *A* Acute coronary syndrome, *B* Back pain

For the indication "traumatic pain," the most commonly provided opioid analgesics are fentanyl (70.1%), piritramide (46.8%), morphine (24.7%), sufentanil (19.5%), and nalbuphine (9.1%). Buprenorphine can only be administered in one of the respondent service area. Among the non-opioid analgesics used to treat traumatic pain, (es)ketamine (90.9%), metamizole (33.7%), and paracetamol (32.4%) are leading; ibuprofen (9.1%), COX2 inhibitors (2.6%), and diclofenac (1.3%) are less commonly provided.

For visceral non-colic pain, morphine (53.2%), fentanyl (33.8%), piritramide (32.5%), sufentanil (13%), nalbuphine (9.1%), and buprenorphine (2.6%) are used. In the non-opioid group, metamizole (68.8%), paracetamol (45.5%), (es)ketamine (12.9%), ibuprofen (3.9%), and the parasympatolytic butylscopolamine (5.2%) are available for this indication.

For use in visceral colic pain, morphine (33.8%) and fentanyl (31.2%), followed by piritramide (29.9%), sufentanil (11.7%), nalbuphine (9.1%), and buprenoprhine (3.9%) are stocked in most ambulance service areas. The non-opioids metamizole (70.1%), paracetamol (33.8%), (es)ketamine (7.8%), and ibuprofen (3.9%) are available for the indication of visceral colic pain. Butylscopolamine is the second most commonly provided agent after metamizole for this indication at 55.8%.

For acute coronary syndrome, the opioids provided in descending frequency are morphine (85.7%), fentanyl (15.6%), nalbuphine (9.1%), sufentanil (5.2%), and piritramide (3.9%). In the non-opioid group, metamizole (10.4%) and paracetamol (10.4%) are the most commonly available.

The opioids used to treat back pain were fentanyl (46.8%), piritramide (40.0%), morphine (28.6%), sufentanil (13.0%), nalbuphine (9.1%), tramal (2.6%), and buprenorphine (1.3%). (Es)Ketamine (37.7%), metamizole (41.6%), paracetamol (29.9%), ibuprofen (15.6%), COX-2 inhibitors (2.6%), and diclofenac (1.3%) are used from the non-opioid group.

The use of inhalative analgesia with Methoxyflurane or LIVOPAN® (50% N2O / 50% O2) was reported once each.

### Independent therapy by emergency paramedics

The independent application of analgesics by paramedics without the need for an emergency physician’s request is carried out in some EMS areas. Piritramide (11.7%), morphine and nalbuphine (7.8% each) are the opioids most commonly approved for independent administration; Fentanyl may be applied independently in two ambulance service areas (2.6%). Of the non-opioid analgesics, (es)ketamine (42.9%), paracetamol (36.4%), metamizole (22.1%), ibuprofen (15.6%), COX-2 inhibitors (2.6%), and the parasympatolytic butylscopolamine (29.9%) may be used independently by emergency paramedics.

The initiation of pain therapy by ambulance personnel with subsequent request of an emergency physician to the scene of the emergency is most common for morphine (14.3%), fentanyl (9.1%), nalbuphine (2, 6%), and piritramide (1.3%), and in the non-opioid group for (es)ketamine (31.2%), paraetamol (16.9%), metamizole (13.0%), ibuprofen (2.6%), and butylscopolamine (15.6%) (Table 2).

**Table 2:** Application of analgesics by emergency paramedics and emergency physicians

Analgesics	Application of analgesics by		
	Paramedics independently	Paramedics with subsequent physician to attend	Only by physician
Piritramide	*N* = 9 (11,7%)	*N* = 1 (1,3%)	*N* = 25 (32,5%)
	*R* = 158 (16%)	*R* = 9 (0,9%)	*R* = 308 (31,1%)
Morphine	*N* = 6 (7,8%)	*N* = 11 (14,3%)	*N* = 40 (51,9%)
	*R* = 133 (13,4%)	*R* = 109 (11%)	*R* = 588 (59,4%)
Nalbuphine	*N* = 6 (7,8%)	*N* = 2 (2,6%)	*N* = 1 (1,3%)
	*R* = 67 (6,8%)	*R* = 32 (3,2%)	*R* = 6 (0,6%)
Fentanyl	*N* = 2 (2,6%)	*N* = 7 (9,1%)	*N* = 43 (55,8%)
	*R* = 30 (3%)	*R* = 114 (11,5%)	*R* = 528 (53,4%)
Sufentanil	0	0	*N* = 12 (15,6%)
			*R* = 283 (28,6%)
Tramal	0	0	*N* = 2 (2,6%)
			*R* = 6 (0,9%)
Buprenorphine	0	0	*N* = 2 (2,6%)
			*R* = 17 (1,7%)
Ketamine	*N* = 33 (42,9%)	*N* = 24 (31,2%)	*N* = 8 (10,4%) R = 148 (15%)
	*R* = 516 (52,1%)	*R* = 303 (30,6%)	
Paracetamol	*N* = 28 (36,4%)	*N* = 13 (16,9%)	*N* = 4 (5,2%)
	*R* = 161 (16,3%)	*R* = 170 (17,2%)	*R* = 85 (8,6%)
Metamizole	*N* = 17 (22,1%)	*N* = 10 (13%)	*N* = 21 (27,2%)
	*R* = 222 (22,4%)	*R* = 96 (10%)	*R* = 414 (41,9%)
COX-2-inhibitors	*N* = 2(2,6%)	0	*N* = 2 (2,6%)
	*R* = 16 (1,6%)		*R* = 63 (6,4%)
Ibuprofen	*N* = 12 (15,6%)	*N* = 2 (2,6%)	*N* = 2(2,6%)
	*R* = 194 (19,6%)	*R* = 28 (2,8%)	*R* = 9 (0,9%)
Diclofenac	0	0	*N* = 1 (1,3%)
			*R* = 6 (0,6%)
Butylscopolamine	*N* = 23(29,9%)	*N* = 12 (15,6%)	*N* = 9 (11,7%)
	*R* = 278 (28,1%)	*R* = 153 (15,5%)	*R* = 212 (21,4%)

*N* number of responders, *R* number of rescue stations

There were no significant differences in the approval of stand-alone analgesic therapy by emergency paramedics for the distinguishing criteria of ambulance service areas such as authorities responsible, size (number of stations…), geography (rural, urban, mixed).

Only the subgroup analysis showed a weak significance (*p* = 0.049/Mann–Whitney U test) for the approval of the substance piritramide in favor of the ambulance service areas with a higher number of ambulance stations (piritramide group versus non-piritramide group with *n* = 8/M 19.75/ SD 15.02 and *n* = 67/M 12.4/ SD 10.23, respectively). When comparing the number of EMS physician stations and the clearance of piritramide therapy for emergency paramedics (piritramide group: *n* = 8/M 7.25 / SD 2.92; non-piritramide group: *n* = 68/M 4.99 / SD 3.61), this association was even more significant (*p* = 0.015/Mann–Whitney U test).

In larger EMS areas (higher number of rescue stations) morphine was authorized for paramedics more frequently than in smaller ones (22.17 vs 12.41), however due to the standard deviation (18.51 vs 9.87) the difference was not significant (*p* = 0.269).

The majority of respondents (53.2%) reported "(in)security in the question of legitimacy when using narcotics" as a reason against the authorization of the independent use of analgesics by paramedics.

The other reasons were lack of experience or confidence on the part of the paramedics in dealing with analgesics (36.4%), immediate availability of an emergency physician (22.1%), lack of special knowledge of the part of the paramedics (19.5%), resistance of individual paramedics to taking on physician competences (16.9%), uncritical indication (16.9%) and fear of an increase in the frequency of side effects (9.1%). 19.5% of participants cited no reasons for opposing the expansion of emergency paramedic authority.

The problem of legal uncertainty is perceived significantly more frequently by MDES who had not approved the administration of opioids by paramedics (*n* = 35/66.0% versus *n* = 6/30%, *p* = 0.006).

The other reasons listed against the approval of stand-alone therapy were not significant (Table 3).

**Table 3:** Reasons against the approval of the independent use of painkillers by ambulance staff

			Opioids	Non-Opioids
**Reasons against the approval of the independent use of painkillers by emergency paramedics**	Yes	No	*p*-value Approved: *n* = 20 Not approved: *n* = 53	*p*-value Approved: *n *= 43 Not approved: *n* = 30
Emergency physician (almost) always available very quickly	17 (22,1%)	56 (72,7%)	*P* = 0,303	*P* = 0,257
The issues of legal (in)certainty in the use of narcotics by emergency paramedics	41 (53,2%)	32 (41,6%)	*P* = 0,006	*P* = 0,020
Indication by emergency paramedics is too uncritical	13 (16,9%)	60 (77,9%)	*P* = 0,284	*P* = 0,683
Lack of expertise of emergency paramedics	15 (19,5%)	58 (75,3%)	*P* = 0,171	*P* = 0,923
Lack of experience/safety in the use of analgesics	28 (36,4%)	45 (58,4%)	*P* = 0,367	*P* = 0,465
Fear of an increase in side effects	7 (9,1%)	66 (85,7%)	*P* = 0,942	*P* = 0,001
Resistance of individual emergency paramedics against the "assumption of medical activity"	13 (16,9%)	60 (77,9%)	*P* = 0,764	*P* = 0,683
There are no reasons	15 (19,5%)	58 (75,3%)		

Significantly more often (*p* = 0.001/ Pearson chi-square test), MDES feared an increase in adverse events with non-opioids if they themselves had not approved these medications (Table 3).

### Substance groups and dosages for emergency paramedics (RDE)

The dosage for intravenous use of morphine by emergency paramedics lies initially predominantly by 1-2 mg (71.4%), 1-2 mg for the repetition (69.2%) and is limited in most cases at 10 mg (75%).

The intravenous administration of fentanyl is an initial dose of 0.05–0.1 mg (100%) and a repeat dose of 0.05–0.1 mg (100%) to a maximum of 0.2 mg (66.6%).

When piritramide is administered intravenously, the initial dose is 1-4 mg (66.6%), in repetition also 1-4 mg (100%) and at the maximum 7.5 mg (57.1%) are administered.

**Fig. 1:**
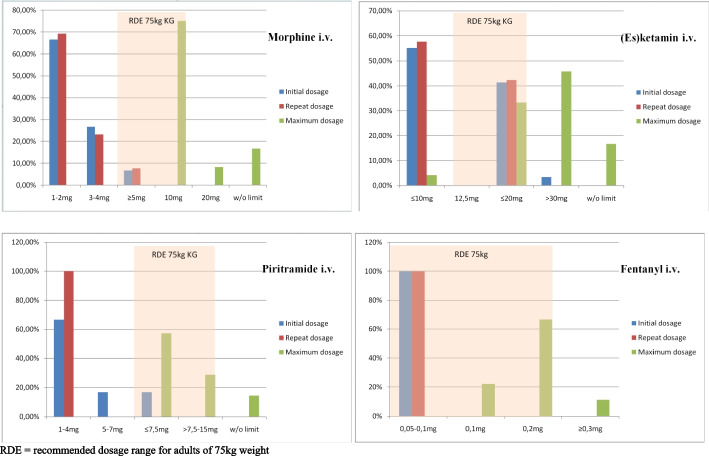
The dosages of important analgesics approved for emergency paramedics. RDE = recommended dosage range for adults of 75 kg weight

For the non-opioid analgesic esketamine, the most common initial intravenous dose is up to 10 mg (55.2%) and up to 20 mg (41.4%). The maximum dosage of esketamine is reported to be 20 mg (33.3%) or more than 30 mg (45.8%) (Fig. 1).

### Assessments on the use of analgesics by emergency paramedics

The assessment of the approval of the use of analgesics for the paramedics is not dependent on type and size of the rescue service area.

There was also a positive response of the MDES for the independent use of opioids by paramedics, with 90.7% in rescue service areas in which approval had not recently been granted.

For non-opioids, it was found that out of 30 MDESs who did not give clearance for stand-alone therapy by emergency paramedics, 80% still considered it useful (*p* = 0.002/ Pearson Chi-square test).

As with the evaluation of these procedures, a positive assessment (78.4%) is also shown with regard to an improvement in the quality of patient care. The feedback did not depend on whether specific procedures were authorized.

Regarding the assessment of analgesic use, the majority of respondents (78.4%) expect an improvement (55.8%) or partial improvement (28.6%) in patient care as a result of the expansion of competences for emergency paramedics.

### Training

Almost half (49.4%) of the survey respondents reported that paramedics are required to undergo special training before they are able to independently administer analgesics. Emergency physicians (42.9%), medical directors of emergency medical services 39%), teaching paramedics (39%) and practice supervisors (35.1%) provided this training. 79.1% of the respondents stated that they offer a refresher course, which must be completed annually in 59.7% and every two years in 9.1% of rescue service areas.

## Discussion

In prehospital emergency medicine, the high prevalence of pain (up to 70%) is a problem that is still not sufficiently solved despite the availability of analgesics [12–15].

The structures of the emergency medical services and their medical management in Germany vary greatly according to the responsibilities of the federal states and the state laws.

In particular, the size of the areas of responsibility of the MDES and the number of callouts differ considerably, the range of responsibility vary from a county with a few ambulances/EMS physician stations to supra-regional responsibility for the EMS of large cities / wide regions or even entire federal states. The "professional relationship" of the MDES also depends on this (full-time, function in addition to work in the hospital), but this was not one of the aims of this study.

Despite the high number of participants in the survey, the "data quality per federal state" was partly insufficient. Concerns had been expressed about data security because in federal states with only one or a few MDES, the results could be assigned to the participating persons.

The actuality and importance of the topic "analgesia in the rescue medical services in Germany" is very high due to changes in the education of emergency paramedics.

Undertreatment of patients with acute pain in emergency services can be linked to the selection of substances by paramedics or emergency physicians (often potency and dosage too low) [7, 14, 19] as well as the treatment of patients in situations where the emergency physician is delayed or unavailable [6, 20].

Morphine (98.7%) and fentanyl (85.7%), highly effective and frequently used µ-agonists in hospitals, are also available in most EMS vehicles. The substances (es)ketamine (98.7%) and metamizole (88.3%) as well as the spasmolytic butylscopolamine (81.8%) are the most frequently available non-opioid analgesics. The analgesically active substances available in the emergency medical services are drugs whose first use dates back between 60 (ketamine) and over 200 years (morphine). Despite newer developments (sufentanil…), it is evident that the "tried and tested" is trusted.

In the case of severe pain (Numeric Rating Scale 9 to 10), the weaker analgesics cannot be used, at least for intra-hospital anaesthesia / analgesia. An exception here is (es)ketamine (in combination with midazolam). The working group of Jabourian et al. was able to show the effectiveness and safety of this therapy when paramedics used ketamine according to a defined scheme in 368 patients [10].

Sufficient analgesia with (es)ketamine can be achieved with a lower risk of respiratory depression. However, in contrast to opioids, there is no possibility of antagonisation, so that the effect cannot be reversed if the borderline between analgesia and anaesthesia is crossed. In this respect, it may also be necessary to secure the airway by endotracheal intubation for (es)ketamine, as with the use of strong opioids (fentanyl, morphine, sufentanil) [15, 16]. In a review article by Sobieraj et al., the quality of analgesia was compared between opioids and (es)ketamine, showing fewer side effects for opioids compared to (es)ketamine with equivalent pain reduction. However, the authors point out that the overall evidence base for this question is poor [21].

A working group led by Shackelford compared the safety of opioids and (es)ketamine during the transport of a total of 119 injured patients from the scene to the hospital; there was no difference in the safety of the use of both groups of medication [22].

The colleagues Häske et al. conducted a meta-analysis of existing studies on prehospital analgesia in traumatised patients, in which 10 randomised, controlled and observational studies were evaluated. Here, too, there was no difference in the effectiveness of pain treatment between the substances fentanyl, morphine and (es)ketamine [23].

In the final consequence, the expected or achieved pain reduction in the case of the most severe pain for sufficiently effective substances is thus to be expected with the same side effects [15, 16].

For the treatment of acute coronary syndrome, the majority of colleagues provide morphine (85.7%). Although textbooks and numerous studies refer to a beneficial effect of morphine in ACS, an advantage of morphine over other potent opioids for this indication has not been proven [24–26].

In Germany, the inhaled analgesics methoxyflurane “Penthrox” (1%) and nitrous oxide/O2 “Livopan” (1%) are rather "exotic". Methoxyflurane has been widely used by inhaler in Australia and New Zealand for about 30 years and is only available in Europe in Belgium, France, Ireland, England and Switzerland [27]. However, the lack of experience in Germany is no reason to fundamentally reject a method that obviously works in other health care systems.

In recent years, the competencies of non-physician emergency medical services personnel in Germany have been expanded [4]. The independent application of analgesics by emergency paramedics is thus possible within the framework of the regional algorithms.

In the past, "curative measures" in emergency medical services were reserved for licensed physicians. This implied legal certainty. While compliance with medical standards could be questioned, the "legitimacy" of the measures could not.

Due to the Emergency Paramedic Act, there is a need in Germany to define the framework for the performance of "curative measures" and medical procedures by non-medical personnel. It is therefore necessary to consider to what extent and in which areas these can be carried out by emergency paramedics and which prerequisites are necessary for this. This also applies to the use of painkillers.

However, it is crucial and necessary, from the patient's point of view, to diagnose and treat the most severe pain as quickly and effectively as possible.

In other medical systems (France, Switzerland, USA…), parts of prehospital emergency care, including analgesia, have already been provided by non-physician ambulance staff for some time [28].

In the studied collective, independent analgesic therapy by emergency paramedics is approved to a significant extent only for non-opioids, most frequently (es)ketamine (42.9%), paracetamol (36.4%), the parasympatolytic buscopan (29.9%) and metamizole (22.1%) may be administered. Stand-alone analgesic therapy with opioids is rare (morphine: 22%). Relatively weakly effective substances (with few side effects) such as piritramide and nalbuphine (synthetic opioid, not listed in the national narcotic act) are also mentioned.

Why substances with high analgesic potential (µ-agonists such as fentanyl) may be used less frequently by emergency paramedics can only be conjectured.

The risk of feared respiratory depression as a threatening side effect is lower with non-opioids. However, in studies with large patient populations, an accumulation of respiratory disturbances did not occur when fentanyl was administered by trained paramedics under defined rules of use (pain measurement, dosages…). In no case was it necessary to secure the airway or only in exceptional cases to antagonise the patient. As a rule, addressing the patient (request to breathe) was sufficient [6, 7, 19].

Greb et al. and Kill et al. have already reported on the safe use of morphine by specially trained paramedics in projects in Germany [29, 30]. The working group of Häske et al. compared the safety and effectiveness of analgesic therapy between specially trained paramedics and emergency physicians; no significant difference was found [31].

The reasons for the relatively rare approval of opioids for use by emergency paramedics in Germany are mainly due to legal (in)certainty (53.2%). This problem is perceived significantly more often by medical directors who had not approved an opioid administration by emergency paramedics (66.0% versus 30.0%, *p* = 0.006). From August 2023, with the amendment of the Narcotics Act, the use of opioids by emergency paramedics will be permitted under defined and regionally approved algorithms [5].

The reasons for the deficits in prehospital analgesia are similar in many countries [9]. The choice of substances and dosages is often made under the aspect of avoiding potentially threatening side effects. In particular, too low doses lead to insufficient pain reduction and thus to rejection of the substances [19].

This study also showed that the doses of analgesics approved for emergency paramedics were in most cases significantly below the recommendations for adults (RDE) [32] (Fig. 1).

Other causes of oligoanalgesia have been identified as low initial NACA and NRS scores and certain CNS and gynaecological disorders [14].

Overall, the lack of national guidelines for prehospital analgesia leads to uncertainties in the creation of algorithms [9, 32]. In perspective, telemedicine could be an improvement with regard to therapy safety, 88.4% of the MDES see it as useful. Large telemedicine centres report good experiences with regard to telemedicine-guided analgesia [17, 18, 33]. However, technical (network density, …) and structural questions (who can be reached…) still have to be answered so that this procedure can be used nationwide.

## Limitations

The 989 ambulance stations covered in this survey by the medical directors represent only a part of the entire ambulance service in Germany. The exact number of rescue stations is not known due to the lack of central registers.

However, due to this high number and the coverage of almost all federal states, it seems possible to draw conclusions about the ambulance service in Germany as a whole.

In the development of the questionnaire, some detailed questions were omitted in the validation process. The answering of complex questions, e.g. on dosage regimens…was partly not complete, here the time frame was obviously exceeded.

A survey on the availability of certain medicines has only limited information on the actual use (and consumption) of these substances.

## Conclusion

The majority of MDES consider the application of opioids by paramedics to be useful.
Many MDES saw the lack of legal certainty as a major obstacle to the approval of opioid administration by emergency paramedics.
For prehospital analgesia, the creation of a guideline as a basis for regional algorithms (pain measurement, selection and dosage of substances…) would make sense.
There are no significant differences in prehospital analgesia between rural and urban regions.
(Es)Ketamine is often used as a potent analgesic and is cleared for use by emergency paramedics more frequently and in higher doses than fentanyl or morphine.

## Data Availability

The datasets used and/or analysed during the current study are available from the corresponding author on reasonable request.

## Abbreviations

EA: Emergency ambulances
EMS: Emergency medical service
EPV: Emergency physician vehicles
MDES: Medical directors of the emergency services
NOA: Non-opioid analgesics
RDE: Recommended dosage for adults of 75 kg weight

## References

[1] Studnek JR, et al. The association between patients’ perception of their overall quality of care and their perception of pain management in the prehospital setting. Prehosp Emerg Care. 2013;17(3):386–91. 23611142 10.3109/10903127.2013.764948

[2] Galinski M, et al. Prevalence and management of acute pain in prehospital emergency medicine. Prehosp Emerg Care. 2010;14(3):334–9. 20507221 10.3109/10903121003760218

[3] Friesgaard KD, et al. Acute pain in the prehospital setting: a register-based study of 41.241 patients. Scand J Trauma Resusc Emerg Med. 2018;26(1):53. 29970130 10.1186/s13049-018-0521-2 PMC6029421

[4] Das Notfallsanitätergesetz vom 22. Mai 2013 (BGBl. I S. 1348), zuletzt geändert durch Beschlussempfehlung und Bericht des Ausschusses für Gesundheit (14. Ausschuss) v. 21.6.2023 (BGBl. I S. 1174) https://www.gesetze-im-internet.de/btmg_1981/index.html#BJNR106810981BJNE000916116 and https://dserver.bundestag.de/btd/20/073/2007397.pdf.

[5] Gesetz über den Verkehr mit Betäubungsmitteln (Betäubungsmittelgesetz - BtMG). "Betäubungsmittelgesetz in der Fassung der Bekanntmachung vom 1. März 1994 (BGBl. I S. 358), zuletzt durch Beschlussempfehlung und Bericht des Ausschusses für Gesundheit (14. Ausschuss) v. 21.6.2023 geändert. Available from: https://www.gesetze-im-internet.de/btmg_1981/index.html#BJNR106810981BJNE000916116 and https://dserver.bundestag.de/btd/20/073/2007397.pdf.

[6] Scharonow M, et al. Project for the introduction of prehospital analgesia with fentanyl and morphine administered by specially trained paramedics in a rural service area in Germany. J Pain Res. 2017;10:2595–9. 29158691 10.2147/JPR.S151077 PMC5683795

[7] Friesgaard KD, et al. Efficacy and safety of intravenous fentanyl administered by ambulance personnel. Acta Anaesthesiol Scand. 2016;60(4):537–43. 26612100 10.1111/aas.12662

[8] Schempf B, Casu S, Haske D. Prehospital analgesia by emergency physicians and paramedics: comparison of effectiveness. Anaesthesist. 2017;66(5):325–32. 28258297 10.1007/s00101-017-0288-2

[9] Dissmann PD, et al. A review of the burden of trauma pain in emergency settings in Europe. Pain Ther. 2018;7(2):179–92. 29860585 10.1007/s40122-018-0101-1 PMC6251834

[10] Jabourian A, et al. Evaluation of Safety and Efficacy of Prehospital Paramedic Administration of Sub-Dissociative Dose of Ketamine in the Treatment of Trauma-Related Pain in Adult Civilian Population. Cureus. 2020;12(8): e9567. 32782893 10.7759/cureus.9567 PMC7411289

[11] Kiavialaitis GE, et al. Clinical practice of pre-hospital analgesia: an observational study of 20,978 missions in Switzerland. Am J Emerg Med. 2020;38(11):2318–23. 31785972 10.1016/j.ajem.2019.10.033

[12] Berben SA, et al. Prevalence and relief of pain in trauma patients in emergency medical services. Clin J Pain. 2011;27(7):587–92. 21505324 10.1097/AJP.0b013e3182169036

[13] Oberholzer N, et al. Factors influencing quality of pain management in a physician staffed helicopter emergency medical service. Anesth Analg. 2017;125(1):200–9. 28489643 10.1213/ANE.0000000000002016

[14] Helm M, et al. Oligoanalgesia in patients with an initial Glasgow Coma Scale Score >/=8 in a physician-staffed helicopter emergency medical service: a multicentric secondary data analysis of >100,000 out-of-hospital emergency missions. Anesth Analg. 2020;130(1):176–86. 31335406 10.1213/ANE.0000000000004334

[15] Hebsgaard S, Mannering A, Zwisler ST. Assessment of acute pain in trauma-A retrospective prehospital evaluation. J Opioid Manag. 2016;12(5):347–53. 27844474 10.5055/jom.2016.0351

[16] Hollis GJ, et al. Prehospital ketamine use by paramedics in the Australian Capital Territory: A 12 month retrospective analysis. Emerg Med Australas. 2017;29(1):89–95. 27699989 10.1111/1742-6723.12685

[17] Brokmann JC, et al. Analgesia by telemedically supported paramedics compared with physician-administered analgesia: a prospective, interventional, multicentre trial. Eur J Pain. 2016;20(7):1176–84. 26914284 10.1002/ejp.843

[18] Lenssen N, et al. Quality of analgesia in physician-operated telemedical prehospital emergency care is comparable to physician-based prehospital care - a retrospective longitudinal study. Sci Rep. 2017;7(1):1536. 28484212 10.1038/s41598-017-01437-5 PMC5431537

[19] Friesgaard KD, et al. Prehospital intravenous fentanyl administered by ambulance personnel: a cluster-randomised comparison of two treatment protocols. Scand J Trauma Resusc Emerg Med. 2019;27(1):11. 30732618 10.1186/s13049-019-0588-4 PMC6367789

[20] Schaller SJ, et al. Differences in pain treatment between surgeons and anaesthesiologists in a physician staffed prehospital emergency medical service: a retrospective cohort analysis. BMC Anesthesiol. 2019;19(1):18. 30704401 10.1186/s12871-019-0683-0 PMC6357417

[21] Sobieraj DM, et al. Comparative effectiveness of analgesics to reduce acute pain in the prehospital setting. Prehosp Emerg Care. 2020;24(2):163–74. 31476930 10.1080/10903127.2019.1657213

[22] Shackelford SA, et al. Prehospital pain medication use by U.S. Forces in Afghanistan. Mil Med. 2015;180(3):304–9. 25735021 10.7205/MILMED-D-14-00257

[23] Haske D, et al. Analgesia in patients with trauma in emergency medicine. Dtsch Arztebl Int. 2017;114(46):785–92. 29229039 10.3238/arztebl.2017.0785 PMC5730701

[24] McCarthy CP, et al. The on- and off-target effects of morphine in acute coronary syndrome: a narrative review. Am Heart J. 2016;176:114–21. 27264228 10.1016/j.ahj.2016.04.004

[25] Ibrahim K, et al. Fentanyl delays the platelet inhibition effects of oral ticagrelor: full report of the PACIFY randomized clinical trial. Thromb Haemost. 2018;118(8):1409–18. 29972861 10.1055/s-0038-1666862 PMC6202927

[26] Senguttuvan NB, et al. Comparison of the effect of Morphine and Fentanyl in patients with acute coronary syndrome receiving Ticagrelor - The COMET (Comparison Morphine, Fentayl and Ticagrelor) randomized controlled trial. Int J Cardiol. 2021;330:1–6. 33600846 10.1016/j.ijcard.2021.02.037

[27] Porter KM, et al. Management of trauma pain in the emergency setting: low-dose methoxyflurane or nitrous oxide? A systematic review and indirect treatment comparison. J Pain Res. 2018;11:11–21. 29302193 10.2147/JPR.S150600 PMC5741984

[28] von Vopelius-Feldt J, Wood J, Benger J. Critical care paramedics: where is the evidence? A systematic review. Emerg Med J. 2014;31(12):1016–24. 24071949 10.1136/emermed-2013-202721

[29] Kill, C., Greb, I., Wranze, E. et al. , Kompetenzentwicklung im Rettungsdienst. Notfall Rettungsmed 04/2007.

[30] I. Greb, E.W., H. Hartmann, H. Wulf, C. Kill, Analgesie beim Extremitätentrauma durch Rettungsfachpersonal. Daten zu Sicherheit und Wirksamkeit bei präklinischer Morphingabe. Notfall Rettungsmed, 02/2011.

[31] Haske D, et al. Prehospital analgesia performed by paramedics: quality in processes and effects under medical supervision. Anaesthesist. 2014;63(3):209–16. 24562597 10.1007/s00101-014-2301-3

[32] Flemming A, Adams HA. Analgesia, sedation and anaesthesia in emergency service. Anaesthesiol Reanim. 2004;29(2):40–8. 15168940

[33] Gnirke A, et al. Analgesia in the emergency medical service: comparison between tele-emergency physician and call back procedure with respect to application safety, effectiveness and tolerance. Anaesthesist. 2019;68(10):665–75. 31489458 10.1007/s00101-019-00661-0

