# Correction: Application of analgesics in emergency services in Germany: a survey of the medical directors

**DOI:** 10.1186/s12873-025-01221-z

**Published:** 2025-04-23

**Authors:** Signe Vilcane, Olga Scharonow, Christian Weilbach, Maximilian Scharonow

**Affiliations:** 1 Department of Anaesthesiology, Intensive Care Medicine, Emergency Medicine and Pain Therapy, St. Josefs-Hospital Cloppenburg, Academic Teaching Hospital of the Hannover Medical School (MHH), Krankenhausstrasse, 13, 49661 Cloppenburg, Germany; 2 Department of Internal Medicine, St. Josefs-Hospital Cloppenburg, Academic Teaching Hospital of the Hannover Medical School (MHH), Cloppenburg, Germany

**BMC Emergency Medicine (2023) 23:104**

10.1186/s12873-023-00878-8

Table 1 in the original article has been amended to more suitably display the methods of administration and indications for the analgesics.

